# Interface-Sensitive Charge Storage and Activation Behavior of Mn(1,3,5-Benzenetricarboxylic Acid (BTC))-Derived Mn_3_O_4_/Carbon Cathodes for Aqueous Zinc-Ion Batteries

**DOI:** 10.3390/molecules30122566

**Published:** 2025-06-12

**Authors:** Jieun Lee, Byoungnam Park

**Affiliations:** Department of Materials Science and Engineering, Hongik University, 72-1, Sangsu-dong, Mapo-gu, Seoul 04066, Republic of Korea; leejieun2670@gmail.com

**Keywords:** MOF, ZIB, interface, AC-EPD, charge storage

## Abstract

In this study, we couple precise interface engineering via alternating current electrophoretic deposition (AC–EPD) with performance-enhancing structural transformation via annealing, enabling the development of high-performance, stable, and tunable Mn-based cathodes for aqueous zinc-ion batteries (ZIBs). Using AC–EPD to fabricate Mn(BTC) (BTC = 1,3,5-benzenetricarboxylic acid) cathodes followed by thermal annealing to synthesize MOF-derived Mn_3_O_4_ offers a synergistic approach that addresses several key challenges in aqueous ZIB systems. The Mn_3_O_4_ cathode prepared via AC–EPD from Mn(BTC) exhibited a remarkable specific capacity of up to 430 mAh/g at a current density of 200 mA/g. Interestingly, the capacity continued to increase progressively with cycling, suggesting dynamic structural or interfacial changes that improved Zn^2+^ transport and utilization over time. Such capacity enhancement behavior during prolonged cycling at elevated rates has not been observed in previously reported Mn_3_O_4_-based ZIB systems. Kinetic analysis further revealed that the charge storage process is predominantly governed by diffusion-controlled mechanisms. This behavior can be attributed to the intrinsic characteristics of the Mn_3_O_4_ phase formed from the MOF precursor, where the bulk redox reactions involving Zn^2+^ insertion require ion migration into the electrode interior. Even though the electrode was processed as an ultrathin film with enhanced electrolyte contact, the charge storage remains limited by solid-state ion diffusion rather than fast surface-driven reactions, reinforcing the diffusion-dominant nature of the system.

## 1. Introduction

Studying zinc-ion batteries (ZIBs) is critically important due to their potential to address key limitations of current energy storage technologies [1,2,3,4]. Unlike lithium-ion batteries, ZIBs utilize zinc metal as the anode, which is both abundant and environmentally benign, making them a cost-effective and sustainable alternative for large-scale energy storage. Additionally, zinc-based systems offer inherent safety advantages owing to their aqueous electrolytes and non-flammable nature [5]. This is particularly important for grid-level applications and consumer electronics where thermal stability is essential. Furthermore, ZIBs can operate with high reversibility and fast ion transport in aqueous media, supporting high-rate capability and long cycle life. As the demand for scalable, low-cost, and safe energy storage grows, research into ZIBs plays a pivotal role in enabling the transition toward more sustainable and secure energy systems.

Studying Mn-BTC (BTC = 1,3,5-benzenetricarboxylic acid)-derived Mn_3_O_4_ cathodes for ZIBs offers several significant advantages [6,7]. First, the metal–organic framework (MOF) structure of Mn(BTC) provides a highly porous and uniform precursor that, upon annealing, decomposes into MnO_2_ with a well-controlled morphology and high surface area. This transformation results in MnO_2_ embedded within a carbon matrix, improving both ionic and electronic transport [8,9]. The derived MnO_2_ retains the redox-active properties of manganese, particularly the Mn^3+^/Mn^4+^ couple, which enables efficient and reversible Zn^2+^ insertion and extraction. Compared to pristine Mn(BTC), the annealed MnO_2_ exhibits greater structural and electrochemical stability. One of the main issues with Mn(BTC) is the dissolution of Mn^2+^ during cycling, which causes interfacial instability and capacity fading [10]. In contrast, the MnO_2_ phase formed after annealing is more stable in aqueous ZnSO_4_ electrolytes and shows minimal voltage fluctuation in early cycles, indicating a more robust electrode–electrolyte interface.

Additionally, the in situ formation of conductive carbon during the annealing process enhances the overall electronic conductivity of the composite, overcoming a known limitation of MnO_2_. The resulting MnO_2_–carbon composite thus benefits from both high capacity and improved rate performance. Previous studies have explored MOF-derived MnO or MnO_2_ materials for ZIBs, often relying on conventional synthesis routes such as high-temperature pyrolysis followed by slurry casting with binders and conductive additives. For example, Yang et al. synthesized MnO_2_@nanoporous carbon composites from Mn-MOF-74 and demonstrated promising microwave absorption properties, but their electrochemical applications were not interface-focused and required post-oxidation and structural modification at 800 °C [11]. Similarly, Zhang et al. developed Fe- and Co-based MOF-derived cathodes that showed moderate performance but required complex composite architectures or metallic dopants to improve conductivity and cycling stability [12,13]. Yin et al. reported the synthesis and application of hierarchical spheroidal MnO@C composites, derived from Mn-based MOFs, as cathode materials for aqueous ZIBs. These MnO@C structures feature MnO cores encapsulated within porous carbon shells, strategically engineered to mitigate key challenges associated with Mn-based cathodes, including Mn^2+^ dissolution, limited electrical conductivity, and rapid capacity degradation during cycling [14].

On the other hand, CeO_2_ was explored for the first time as a cathode material in aqueous ZIBs, showing a high initial capacity of 248.9 mAh/g and stable performance (143.8 mAh/g after 500 cycles) [15]. The study revealed that Mn^2+^ dissolution and redeposition are essential for battery operation, with CeO_2_ acting to stabilize and catalyze Mn^2+^. This mechanism is similar to Zn||MnO_2_ systems, suggesting that MnO_2_ itself may not be essential if a suitable host material like CeO_2_ is used, offering new directions for oxide cathode design.

In contrast, our approach circumvents these limitations by directly depositing the Mn(BTC)-derived material onto stainless steel using AC–EPD, eliminating the need for conductive additives or binders that often obscure interfacial behavior. This strategy uniquely positions the electrode as a sensitive platform for probing Mn dissolution—a known challenge in Mn-based ZIB systems that is often overlooked or indirectly assessed in thicker composite electrodes. The ultrathin nature of the film, combined with the controlled deposition conditions of AC–EPD, allows us to isolate and monitor with unprecedented clarity interfacial processes such as early-stage Mn leaching, activation behavior, and structural transformation. Upon annealing, Mn(BTC) is converted into Mn_3_O_4_, forming a MOF-derived Mn_3_O_4_–carbon composite. This transformation improves the structural and chemical stability of the cathode. While pristine Mn(BTC) suffers from dissolution of Mn^2+^ during cycling, the derived MnO_2_ has lower solubility and forms a more electrochemically stable phase, significantly enhancing cycling performance and mitigating initial voltage fluctuations.

Moreover, the annealing process results in the in situ formation of conductive carbon from the organic BTC ligands. This carbon matrix enhances the electrical conductivity of the MnO_2_ composite, which otherwise suffers from poor intrinsic conductivity, and provides interconnected porous pathways that promote rapid Zn^2+^ transport.

Finally, the combination of AC–EPD and annealing enables the creation of a well-defined model system for studying structure–property relationships in MOF-derived cathodes. The as-fabricated structure allows researchers to investigate how interface behavior, phase transformation, and charge storage mechanisms evolve from the pristine MOF to the annealed oxide phase.

The novelty of our research strategy lies in the combination of using an MOF (Mn(BTC)) as a precursor and employing an interface-sensitive, additive-free AC–EPD method to fabricate ultrathin Mn_3_O_4_ cathodes for aqueous ZIBs. To the best of our knowledge, this is the first study to report such a high capacity (~800 mAh/g) in a Mn_3_O_4_ cathode derived from an Mn(BTC) MOF precursor without any conductive additives, while simultaneously offering a mechanistic insight into the electrode–electrolyte interface. Thus, our work introduces a new experimental framework for studying and improving MOF-based ZIB cathodes, bridging fundamental interfacial science with practical device performance.

## 2. Results and Discussion

**Figure 1:**
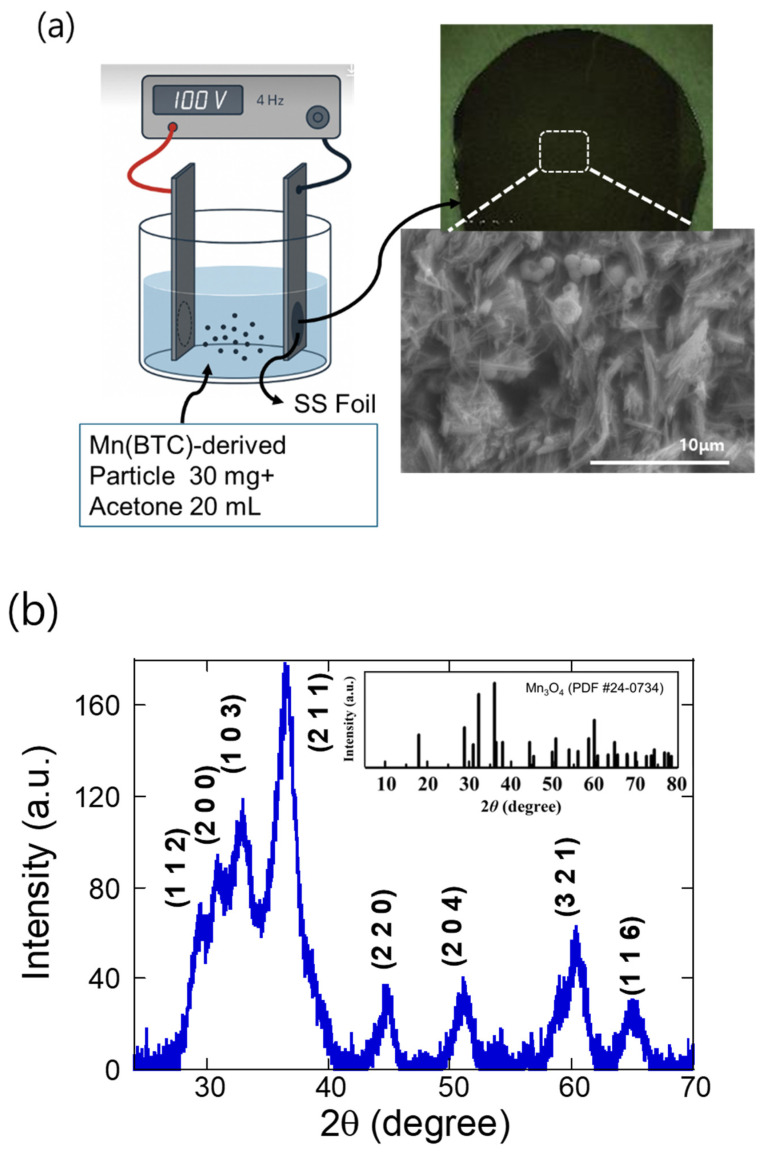
(**a**) Schematic illustration of the AC electrophoretic deposition (AC–EPD) setup used for depositing Mn(BTC)-derived MnO onto stainless steel (SS) foil, accompanied by an optical image of the SS substrate and an SEM micrograph of the deposited MnO film. (**b**) X-ray diffraction (XRD) pattern of the MnO cathode fabricated by AC–EPD, confirming the formation of a crystalline MnO phase.

Figure 1a illustrates the experimental configuration of the alternating current electrophoretic deposition (AC–EPD) system employed in this work. Stainless steel (SS) foils were used as the conductive substrates for film deposition, and an alternating voltage of 100 V at a frequency of 4 Hz was applied to drive the process. This setup enabled the uniform deposition of the Mn(BTC)-derived material across the surface of the SS foil, ensuring consistent film coverage and good interfacial contact. The SEM image shows the surface morphology of MnO_2_ derived from Mn(BTC) after thermal annealing, captured at 5000× magnification. The structure consists of randomly oriented, rod-like, and flake-shaped particles, indicating that the original MOF has transformed into a crystalline MnO_2_ phase while partially retaining a porous, loosely packed texture. The elongated features suggest anisotropic growth during annealing, while the overall morphology remains rough and interconnected. The porous and open structure is favorable for ion transport, making it beneficial for applications such as zinc-ion battery cathodes where high surface area and diffusion accessibility are critical.

The X-ray diffraction (XRD) pattern shown in Figure 1b corresponds to Mn_3_O_4_ and displays well-defined peaks that are characteristic of its tetragonal spinel crystal structure. The major diffraction peaks are indexed to the (112), (211), (220), (204), (321), and (116) planes, which are consistent with the standard JCPDS card No. 24-0734 for Mn_3_O_4_ [10,16].

**Figure 2:**
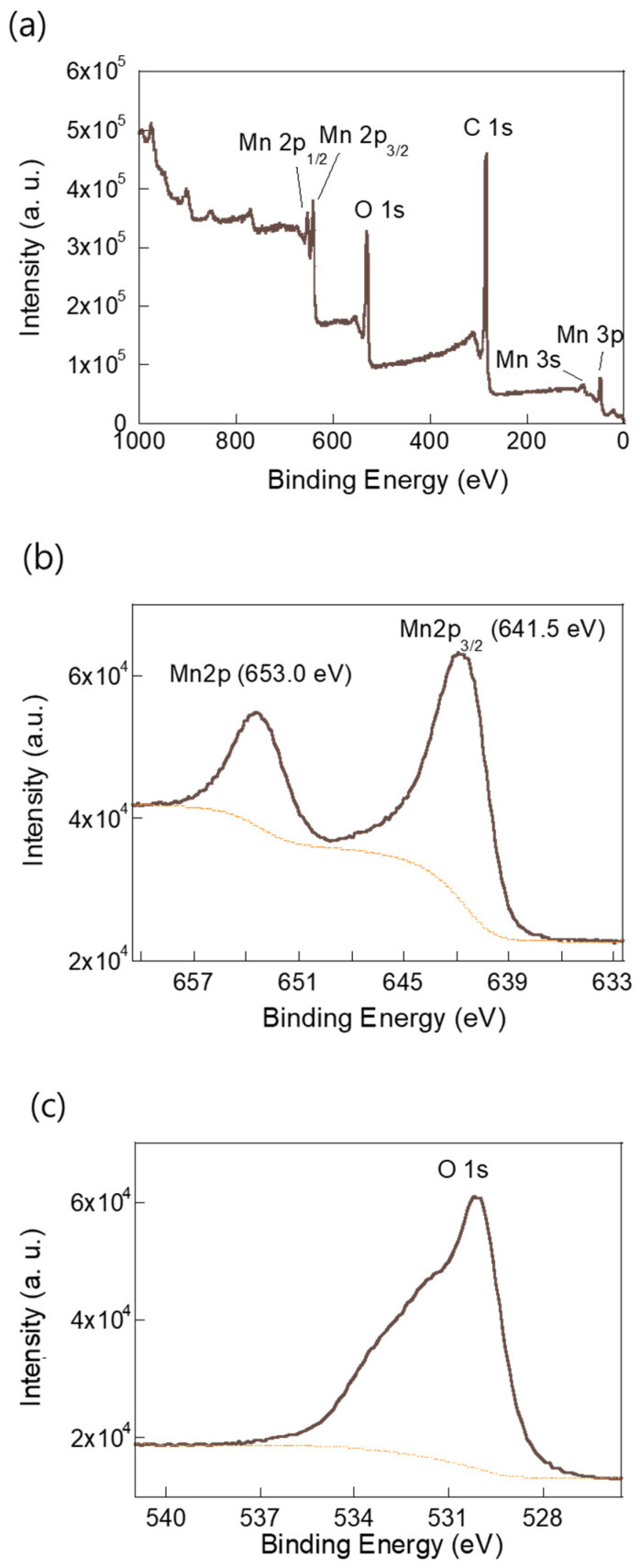
(**a**) X-ray photoelectron spectroscopy (XPS) survey spectrum of the MnO film. (**b**) High-resolution Mn 2p and (**c**) O 1s spectra confirming the Mn and oxygen chemical states.

XPS was employed to investigate the elemental composition and chemical states of the annealed Mn-BTC sample. The survey spectrum (Figure 2a) confirmed the presence of manganese (Mn), carbon (C), and oxygen (O), indicating the compositional purity of the material. As shown in Figure 2b, two prominent peaks at binding energies of 641.5 eV and 653.0 eV correspond to Mn 2p_3/2_ and Mn 2p_1/2_, respectively, confirming the formation of Mn_3_O_4_. Furthermore, the deconvoluted O 1s spectrum in Figure 2c reveals distinct peaks attributed to C–O (531.2 eV), C=O (532.5 eV), and Mn–O (529.8 eV), supporting the presence of both surface functional groups and Mn–O bonding, thereby affirming the structural integrity and surface chemistry of the composite [17,18].

**Figure 3:**
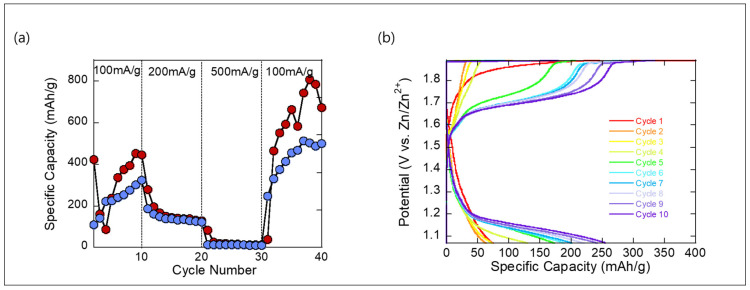
(**a**) Galvanostatic charge–discharge (GCD) curves of the MnO cathode at various current densities Red: charging. Blue; discharging. (**b**) Corresponding voltage profiles for the initial 10 cycles, highlighting the evolution of charge–discharge behavior during early cycling.

The rate capability of the Mn_3_O_4_ cathode was evaluated through GCD measurements and corresponding voltage profiles, as presented in Figure 3a. The GCD plot shows the electrochemical performance of Mn_3_O_4_ obtained by annealing Mn(BTC), tested under varying current densities. In the initial cycles at 100 mA/g, the specific capacity gradually increases, indicating electrochemical activation of the Mn_3_O_4_ electrode, likely due to improved electrolyte infiltration and the formation of electrochemically active sites. When the current density is increased to 200 and then 500 mA/g, the capacity decreases due to kinetic limitations and incomplete utilization of the active material. Notably, when the current is reduced back to 100 mA/g, the capacity not only recovers but significantly increases, reaching values as high as 800 mAh/g. This remarkable recovery suggests that the electrode undergoes structural or interfacial evolution during high-rate cycling, leading to enhanced Zn^2+^ accessibility and improved charge storage performance. The result highlights the high reversibility and activation behavior of the annealed Mn_3_O_4_ electrode.

Interestingly, polarization in the voltage profile was more pronounced at lower current densities and diminished at higher rates. This counterintuitive trend suggests that initial cycling at slower rates may trigger electrochemical or structural activation—possibly related to ion redistribution, redox stabilization, or improved interfacial contact. Once activated, the system exhibits reduced overpotential, even under higher current operation. This behavior highlights the dynamic evolution of the electrode–electrolyte interface and underscores the importance of early-cycle conditioning.

The voltage profiles of the annealed Mn_3_O_4_ electrode over the first 10 cycles in Figure 3b reveal a clear electrochemical activation process, consistent with the GCD results. In the initial cycle, the electrode exhibits low capacity and significant voltage polarization, indicative of sluggish Zn^2+^ ion transport and interfacial resistance. However, as cycling progresses, the charge–discharge curves gradually extend along the capacity axis and the voltage hysteresis narrows, reflecting enhanced reversibility and improved reaction kinetics. The sloping nature of the profiles suggests a solid-solution mechanism for Zn^2+^ insertion and extraction, rather than a distinct phase transformation. This progressive evolution of the voltage profiles supports the capacity increase observed in the GCD plot and highlights the structural stabilization and activation of the Mn_3_O_4_ electrode during early cycling.

**Figure 4:**
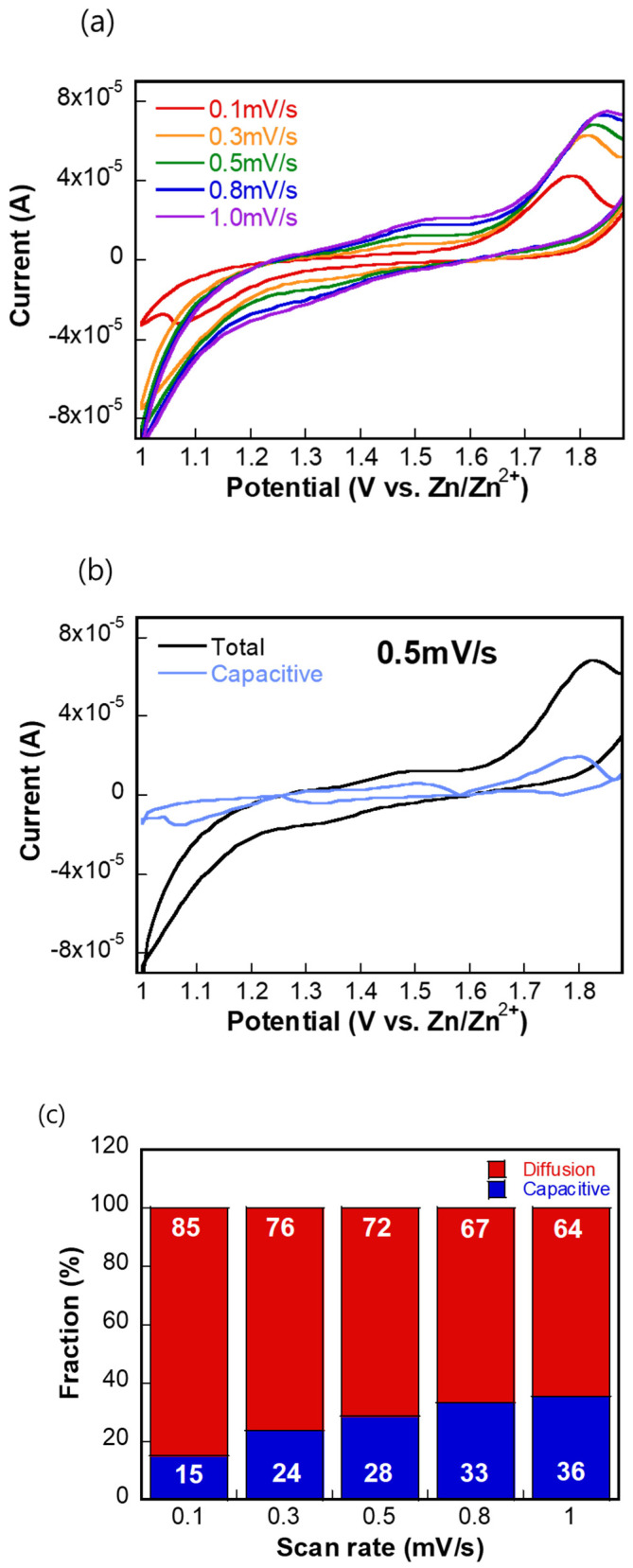
(**a**) Cyclic voltammetry (CV) curves of the MnO cathode at scan rates of 0.3, 0.5, 0.8, and 1.0 mV/s. (**b**) CV curve at 0.5 mV/s showing the contribution of the capacitive processes (blue line) (**c**) Quantitative analysis of the relative contributions of capacitive and diffusion-controlled charge storage mechanisms at different scan rates.

To evaluate the charge storage mechanism of Mn_3_O_4_ derived from annealed Mn(BTC), CV measurements were conducted at various scan rates, and the contributions from diffusion-controlled and capacitive processes were quantitatively analyzed. Figure 4a displays the CV curves obtained at scan rates ranging from 0.1 to 1.0 mV/s. As the scan rate increases, the current response also increases, and the shape of the CV curves gradually shifts—indicating a growing contribution from capacitive processes. This qualitative change suggests a mixed charge storage behavior involving both surface-controlled (capacitive) and bulk diffusion-controlled mechanisms.

To quantify the capacitive contribution, the total current at a given potential *i*(*V*) was deconvoluted into capacitive and diffusion-controlled components using the equation [19,20] *i*(*V*) = *k*_1_*v* + *k*_2_*v*^1/2^*,* where *k*_1_*v* represents the capacitive contribution and *k*_2_*v*^1/2^ corresponds to diffusion-limited processes. Figure 4b shows the result of this analysis at a scan rate of 0.5 mV s^−1^, where the capacitive current (blue) is overlaid on the total current (black). The significant overlap at high potentials suggests that capacitive storage dominates in these regions.

Figure 4c summarizes the fraction of capacitive and diffusion-controlled contributions at each scan rate. At the lowest scan rate (0.1 mV/s), diffusion-controlled processes dominate, contributing 85% of the total current. As the scan rate increases, the capacitive fraction gradually rises, reaching 36% at 1.0 mV/s. This trend is expected, as higher scan rates favor surface-limited reactions due to the reduced time for ion diffusion into the bulk of the material. Overall, this analysis confirms that while Mn_3_O_4_ primarily stores charge via diffusion-controlled redox reactions, capacitive processes become increasingly significant at higher scan rates, contributing to the high-rate performance of the electrode.

The diffusion coefficient of Zn^2+^ ions in the annealed Mn(BTC) electrode (converted to Mn_3_O_4_) was extracted using the Randles–Sevcik equation, which relates the peak current in cyclic voltammetry to the electrochemical and transport properties of the system. Specifically, the anodic peak current obtained at a scan rate of 0.5 mV/s was used in the analysis. The Randles–Sevcik equation for a reversible redox reaction is expressed with the following variables: *i_p_* is the peak current (A); *n* is the number of electrons transferred (assumed to be 2 for Zn^2+^); *A* is the electrode area (1.0 cm^2^); *C* is the concentration of Zn^2+^ in the electrolyte (1 mol/L or 1 × 10^−3^ mol/cm); *ν* is the scan rate (0.5 mV/s); and *D* is the diffusion coefficient (cm^2^/s). Rearranging the equation to solve for *D* and substituting the known values, the diffusion coefficient was calculated to be approximately 1.29 × 10^−11^ cm^2^/s. This value confirms that the Zn^2+^ charge storage process in Mn_3_O_4_ is predominantly diffusion-controlled, consistent with the low *b*-value obtained from the log–log analysis of peak current versus scan rate.

**Figure 5:**
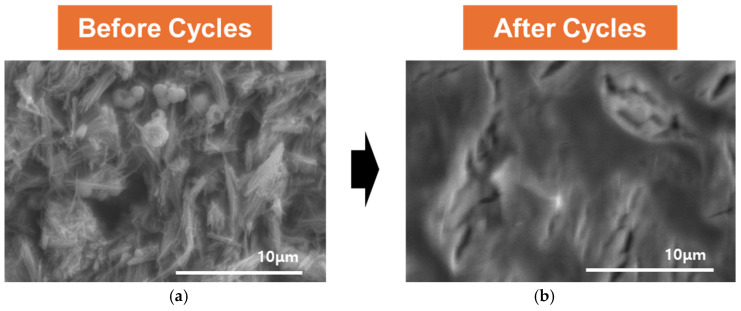
SEM images of the MnO cathode surface (**a**) before and (**b**) after 40 charge–discharge cycles, showing morphological changes and evidence of interfacial degradation or densification upon cycling.

The SEM images in Figure 5 show the morphological evolution of Mn_3_O_4_ derived from Mn-BTC after annealing at 700 °C in a nitrogen atmosphere, comparing the structure before and after electrochemical cycling. Before cycling, the electrode surface exhibits a distinct, textured morphology with well-defined, angular flake-like structures, indicative of a porous and crystalline architecture favorable for ion transport. After cycling, however, the surface undergoes a dramatic transformation, becoming significantly smoother and more compact with the disappearance of the original microstructural features. This change suggests structural collapse and surface densification, likely due to Zn^2+^ insertion/extraction-induced stress, Mn dissolution, and interfacial degradation over repeated cycles. The observed morphological shift underscores the importance of interfacial and structural stability in preserving the electrochemical performance of MOF-derived Mn-based electrodes.

**Figure 6:**
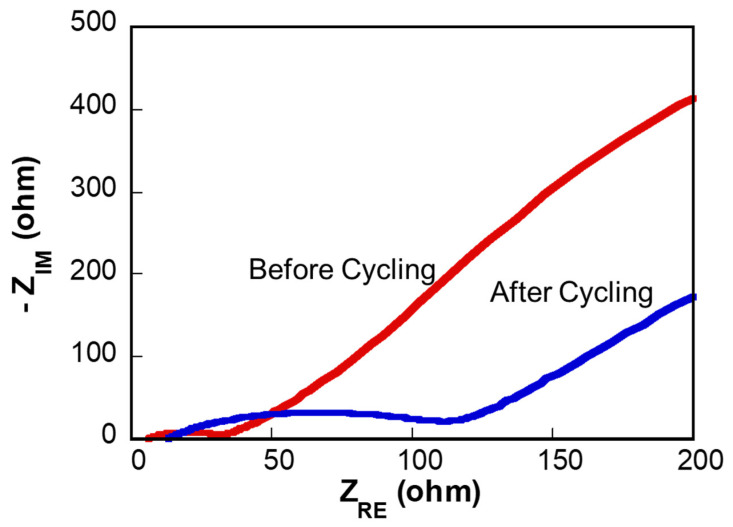
Nyquist plots of the Mn(BTC)-derived Mn_3_O_4_ electrode before (red curve) and after (blue curve) cycling.

EIS analysis revealed changes in charge transfer resistance and diffusion behavior upon cycling, as shown in Figure 6. Before cycling (red curve), the charge transfer resistance was relatively low at approximately 26 Ω, and the diffusion coefficient calculated from the Warburg region was 2.5 × 10^−10^ cm^2^/s. However, after cycling (blue curve), the charge transfer resistance significantly increased to about 102 Ω, yet the diffusion coefficient improved notably to 9.8 × 10^−10^ cm^2^/s. These results suggest cycling-induced structural modifications at the electrode/electrolyte interface, which increased resistance while simultaneously enhancing ionic diffusivity within the electrode material.

In comparison to previously reported ZIBs utilizing MOF-derived Mn-based materials, the Mn_3_O_4_ electrode fabricated in this work via AC–EPD from Mn(BTC) exhibits superior electrochemical performance and interfacial clarity. Many studies have explored MnO and MnO_2_ derived from MOFs such as Mn-MOF-74 or Mn-BDC as cathodes in ZIBs, often requiring high-temperature pyrolysis and incorporation of conductive additives or binders to stabilize the active material. For instance, MnO@C or MnO_2_@C composites prepared from Mn-MOFs have demonstrated moderate capacities in the range of 150–300 mAh/g, but they often suffer from rapid capacity fading due to poor interfacial stability and uncontrolled Mn dissolution during cycling. 

In contrast, our additive-free, ultrathin Mn_3_O_4_ electrode—derived from Mn(BTC) and deposited via AC–EPD—achieves a much higher specific capacity of up to 800 mAh/g at 100 mA/g after electrochemical activation. This enhancement is attributed not only to the intrinsic properties of the MOF precursor but also to the unique advantages of the AC–EPD process, which enables uniform deposition, excellent electrode–electrolyte contact, and interface-dominated electrochemical behavior. The platform’s sensitivity to early-stage Mn dissolution allows for more accurate tracking of degradation mechanisms, a feature that is typically obscured in conventional slurry-coated or bulk-derived electrodes.

Further, Mn_3_O_4_ has a mixed-valent nature (Mn^2+^/Mn^3+^) and surface redox-active sites, which contribute to pseudocapacitive behavior. The surface faradaic reactions (e.g., fast Mn^2+^ ↔ Mn^3+^ transitions) can occur with minimal diffusion of Zn^2+^ into the bulk, especially under low scan rates. This redox activity is often quasi-surface-confined, enabling a high ratio of capacitive-like charge storage. Further, the ultrathin, binder/additive-free film created by AC–EPD exposes more electrochemically accessible surface area, enhancing capacitive contributions. AC–EPD ensures uniform particle distribution and minimal tortuosity, which supports rapid ion transport and suppresses resistive bottlenecks. It avoids buried active sites (common in slurry-cast electrodes), maximizing interfacial redox utilization. The capacitive contribution is inherent to the Mn_3_O_4_ material but is amplified by the interface-sensitive, binder-free AC–EPD electrode structure. In other words, the synergy between material chemistry and architectural control enables high capacitive behavior, especially evident at higher scan rates. Overall, our results suggest that interface-engineered architectures like AC–EPD Mn_3_O_4_ can unlock the full potential of MOF-derived materials, offering a promising route toward high-capacity, stable zinc-ion batteries.

## 3. Experimental Methods

Fabrication of AC–EPD MnO/C Cathode derived from Mn-BTC.

### 3.1. Materials Synthesis

Mn(BTC) was synthesized through a two-step coordination precipitation method. First, 2450 mg of manganese(II) acetate and 600 mg of polyvinylpyrrolidone (PVP) were dissolved in a 1:1 ethanol–water solution (125 mL each) to prepare the metal precursor solution (Solution A). In parallel, 4500 mg of 1,3,5-benzenetricarboxylic acid (H_3_BTC) was dissolved in the same ethanol–water solvent system to form Solution B. Under constant stirring, Solution B was gradually added to Solution A and the resulting mixture was stirred for 10 min, then aged undisturbed for 24 h to allow crystal growth. The precipitated Mn(BTC) product was isolated by centrifugation, thoroughly rinsed with ethanol, and dried at 60 °C. The dried solid was subsequently ground using a ball mill and subjected to thermal annealing at 700 °C for 2 h under a nitrogen atmosphere to induce conversion to MnO_x_, suitable for further electrode fabrication.

### 3.2. Electrode Fabrication by AC–EPD and Electrochemical Characterizations

AC electrophoretic deposition (AC–EPD) was employed to deposit Mn-BTC onto stainless steel (SS) foils using an alternating sinusoidal signal. SS foils were cleaned using acetone–methanol in a sonication bath for 10 min. The deposition was conducted in an acetone-based suspension containing 30 mg of thermally treated Mn-BTC powders dispersed in 20 mL of acetone, followed by ultrasonication for 30 min to ensure uniform dispersion. The AC–EPD process was carried out at an applied voltage of 100 V and a frequency of 4 Hz for 15 min, resulting in a uniform film on the SS substrate. The electrode film had a thickness of approximately 1 μm and a mass loading of 0.3 mg/cm^2^. Thickness was determined using a surface profilometer (Alpha-Step, Torrance, CA, USA). For electrochemical evaluation, CR2032-type coin cells were assembled using Zn foil as the anode, the Mn-BTC-coated SS foil as the cathode, and an aqueous electrolyte composed of 2 M ZnSO_4_ and 0.1 M MnSO_4_ to inhibit further Mn^2+^ dissolution from the electrode.

The electrochemical performance was tested through galvanostatic charge/discharge cycling within a voltage range of 1.0–1.9 V vs. Zn/Zn^2+^, using a battery testing system (BTS, Neware, Shenzhen, China). Current density varied from 100 mA/g to 500 mA/g during cycling. Additionally, cyclic voltammetry (CV) was performed at scan rates of 0.1, 0.3, 0.5, 0.8, and 1.0 mV/s within the same voltage range to assess the redox behavior of the electrodes. To complement the electrochemical analysis, a series of spectroscopic and structural characterizations were conducted on the annealed Mn(BTC) electrodes. X-ray photoelectron spectroscopy (XPS), scanning electron microscopy (SEM), electrochemical impedance spectroscopy (EIS), and X-ray diffraction (XRD) were employed on freshly prepared samples to confirm their phase purity, surface morphology, interfacial properties, and crystallographic structure.

## 4. Conclusions

An ultrathin Mn_3_O_4_ electrode derived from Mn(BTC) was fabricated using an additive-free AC–EPD method to construct a ZIB system with enhanced interface sensitivity. This approach enables precise investigation of interfacial phenomena, particularly Mn dissolution, by minimizing bulk effects and emphasizing surface-driven processes. The resulting platform offers a powerful means to elucidate degradation pathways and provides critical insight into the interfacial instability of MOF-based electrodes. These findings serve as a foundation for developing more robust and long-lasting cathode materials for aqueous zinc-ion batteries. The observed changes in capacity throughout the cycling process are closely linked to the use of the interface-sensitive AC–EPD fabrication method, which enables precise control of the electrode–electrolyte interface. During the initial cycles at 100 mA/g (Cycles 1–15), the specific capacity gradually increases from approximately 100 to 400 mAh/g, reflecting electrochemical activation of the Mn_3_O_4_ structure. This activation is likely due to enhanced electrolyte wetting, interfacial reconstruction, and the gradual formation of electrochemically active regions—all of which are effectively captured in the AC–EPD-processed ultrathin electrode. Upon increasing the current density to 200 and 500 mA/g (Cycles 16–31), the capacity decreases, consistent with kinetic limitations such as sluggish Zn^2+^ diffusion and incomplete utilization of the active material. Notably, when the current density is reduced back to 100 mA/g (Cycles 32–40), the capacity not only recovers but significantly increases to around 800 mAh/g. This dramatic enhancement suggests reversible structural and interfacial evolution, possibly involving the exposure of new active sites or increased surface accessibility—phenomena made evident due to the interfacial sensitivity of the AC–EPD platform. The use of AC–EPD enabled the formation of a dense, binder-free Mn_3_O_4_ electrode with a well-defined interface, which effectively reduced Mn dissolution by minimizing exposed reactive sites and eliminating additive-induced electrochemical inhomogeneity.

## Data Availability

The data presented in this study are available on request from the corresponding author.
